# Relationships between land tenure insecurity, agrobiodiversity, and dietary diversity of women of reproductive age: Evidence from Acholi and Teso subregions of Uganda

**DOI:** 10.1111/mcn.12965

**Published:** 2020-12-21

**Authors:** Beatrice Ekesa, Richard M. Ariong, Gina Kennedy, Mary Baganizi, Ian Dolan

**Affiliations:** ^1^ Bioversity International, Healthy Diets from Sustainable Food Systems Initiative Kampala Uganda; ^2^ Bioversity International, Healthy Diets from Sustainable Food Systems Initiative Rome Italy; ^3^ Trócaire Kampala Uganda

**Keywords:** dietary diversity, land tenure insecurity, species biodiversity, Uganda, women of reproductive age

## Abstract

Land tenure security is central to food security of rural agricultural‐dependent communities, but there is limited evidence linking the state of agrobiodiversity to perception of land tenure security and access to and quality of food eaten. This study explores this relationship using data captured from 1,279 households in Acholi and Teso subregions of Uganda, and the relationships are established using a study sample of 1,227 women of reproductive age (WRA). Sixteen percent of respondents perceived themselves to be land tenure insecure. Although approximately 275 species were reported available for food, household access to a variety of plant and animal species is limited to <10 species by 69% of the study population. Dietary diversity was also low, with 53% of women meeting minimum diet diversity. Evidence from estimation of a generalized Poisson regression reveals that dietary diversity of WRA is consistently, positively correlated with species diversity available for food and negative with land tenure insecurity. A unit increase in species diversity led to 18% increase in dietary diversity of WRAs. Land tenure insecurity was likely to reduce dietary diversity of WRAs by 26% (*p* < .05). Interventions with an aim to increase species diversity can deliver positive dividends for food and nutrition security. Land policy reforms and interventions that strengthen land tenure security for both men and women are likely to contribute positively to dietary diversity leading to improved food and nutrition security of vulnerable communities in rural areas.

**Key messages**

- Despite 16% of households with women of reproductive age (WRA) perceived themselves to be insecure with regards to land tenure security, the studies small holder households in Teso and Acholi subregions of Uganda can access 55 cultivated plant species, eight domesticated animal species, and more than 150 wild plant and animal species for food.
- Almost half of the women (47%) were not meeting their minimum dietary diversity score and majority of those WRA who did not meet the MDD ate more of starchy staples (cereals, roots, tubers, and bananas) and vegetables and a lesser percentage ate pulses, nuts/seeds, dairy, meats, eggs, and vitamin A–rich vegetables and fruits. For all categories, consumption of milk and milk products, vitamin A–rich vegetables and fruits, and eggs was low.
- A unit increase in the number of species accessed for food by WRA is likely to increase their dietary diversity by 18%.
- The perception of being land tenure insecure has a likelihood of reducing diversity of diets consumed by WRA age by 26%.

## 1 INTRODUCTION

One of the greatest global challenges is to secure sufficient and healthy food for all and to do so in a sustainable manner (Burchi, Fanzo, & Frison, 2011). Under global mandate of sustainable development goals, world leaders and proponents of development agreed on agenda 2030 that chiefly aims at achieving sustainable development in all dimensions namely social, economic, and environmental dimensions. Key among the 17 goals is eradication of poverty in all its forms and dimensions and ending hunger and malnutrition through efforts that promote food security and sustainable agriculture to ensure access to safe, nutritious, and sufficient food for all but more so for the world's most vulnerable (United Nations [UN], 2015). The UN targets expect to ensure that all vulnerable men and women have equal rights to economic resources as well as access to basic services, ownership and control over land and other forms of inheritance, natural resources, and technologies. Additionally, it is also recognized that achieving a food secure world requires maintenance of genetic diversity of seed, cultivated plants, farmed and domesticated animals, and their related wild species that form a great part of agricultural biodiversity. Agricultural biodiversity also known as agrobiodiversity is defined as “the variety and variability of plants, animals, and microorganisms at genetic, species, and ecosystem level.” Although most efforts for biodiversity conservation have traditionally aimed at supporting protected areas, the indicated link to food and nutrition security creates the need to investigate participatory models of biodiversity management in agricultural ecosystems that embrace biodiversity for farmers' food and livelihoods (United Nations University [UNU], 2003).

Land is the key foundation for agrobiodiversity. A farmer's perception or sense of security in relation to their land can have positive or negative effect on short‐term decisions and long‐term investments especially in terms of conservation practices and crop and animal choices that later determine nutrition outcomes for subsistence households, where the primary objective for production is household consumption. It influences the extent to which farmers are prepared to invest in improvements in production and land management (International Fund For Agricultural Development, 2015). It is also suggested that tenure security shapes social relations and contributes to social stability—or rather, situations of tenure insecurity contribute to social instability and conflict. Without expounding on the nature of land ownership, land size has a negative correlation with dietary diversity (Hossain, Jimi, & Islam, 2016). The certainty with which dwellers on land have on the stability of their future survival on it without threats from external and/or internal parties is thus pertinent to household livelihood and both agrobiodiversity and dietary diversity. Any factor that undermines ones' confidence of land tenure stability is a precursor for land impermanence syndrome. Land tenure security implies that the farmers or people using and living on a certain piece of land are certain of its ownership status, therefore land impermanence syndrome/land tenure insecurity involves farmer apprehension or uncertainty about the future ownership status on land and leads to disinvestment in an agricultural operation as well as erosion of producer confidence (Parry & Skaggs, 2014). Thus, land impermanence syndrome can undermine planning and encourage speculation according to Parry and Skaggs (2014). In addition, secure access to sufficient land is an important means of achieving food security in poor agrarian land‐scarce societies, and strong tenure security for landowners stimulates investment and efficiency of land use (Holden & Ghebru, 2016).

Despite all the above evidence linking, land tenure security to agrobiodiversity conservation and utilization and food and nutrition security, these kinds of linkages have not been studied extensively in developing countries and especially East Africa; most of the studies like those by M'Kaibi, Steyn, Ochola, and Du Plessis (2016) and Saaka, Osman, and Hoeschle‐Zeledon (2017) have focused on linking production and consumption diversity, and it is no longer enough to keep examining the impact of agricultural interventions on food quality alone while neglecting where the food primarily comes from, land. Moreover, agriculture, land, and nutrition cannot be separated, but agriculture interventions have had gaps in contextualizing nutrition and overly lay emphasis on determining impacts of agricultural interventions on food consumption and diet quality (Herforth & Ballard, 2016).

This study examines the changes in agrobiodiversity for two distinct categories of households: those perceiving themselves secure and those perceiving themselves insecure with regards to land tenure. The study further evaluates the association between the perception regarding land tenure and utilization of agrobiodiversity and the relationship with consumption patterns of vulnerable population groups, particularly women of reproductive age. The entire study areas were affected by the insurgency during the Lord's Resistance Army civil war that lasted 20 years (1985–2005) in northern Uganda and parts of eastern Uganda. Rehabilitation and total recovery of the affected communities are still ongoing in Teso subregion, eastern Uganda, and Acholi subregion in northern Uganda. During insurgency in the two subregions, land owners were displaced from their customary land and forcibly taken to camps by the government as a way of “protection.” However, upon return to their land starting in 2005, the regions that were affected have suffered several land conflicts arising due to contested access to a land and disputes over land boundaries. Boundary disputes are also associated with traditional use of nonpermanent markers such as trees and shrubs which at times are lost because of various factors. Most of the people in Acholi and Teso are poor, and according to Fanzo (2018), the global population most affected by food insecurity and malnutrition includes the poor, rural, isolated, women and children, marginalized, and conflicted. The overriding hypothesis was that the household perception of land tenure insecurity could explain one of the food security dimensions—food quality—described in terms of dietary diversity. The study looks at land beyond its physicality in terms of size to understand how the unobserved fear of land being taken by another party affects the choices with regards to what food commodities the households choose to grow and rear for both food and income and any other practices related to conservation of agrobiodiversity thus influencing food options that in turn can have effect on consumption patterns for women of reproductive age (WRA).

## 2 METHODS

### 2.1 Study area and sampling procedure

This study was conducted in Acholi, which is a subregion in northern Uganda and Teso subregion in eastern Uganda, where both rural subregions suffered the brunt of insurgency from 1985 to 2005. Acholi subregion is predominantly an area occupied by people of Acholi ethnicity who are 4% of Uganda's population, whereas Teso is predominantly an area occupied the Iteso ethnic group and are the fourth largest ethnic group with a share of 8% of Uganda's population (Uganda Bureau of Statistics [UBOS], 2016). Due to insurgency the social and agricultural systems of both Acholi and Iteso people have been disrupted thus affecting what they grow, rear, purchase, sell, and other methods of acquiring food such as animal hunting and trapping and plant gathering that may predicate changes in the current resilience to food shocks compared with the past.

**FIGURE 1:**
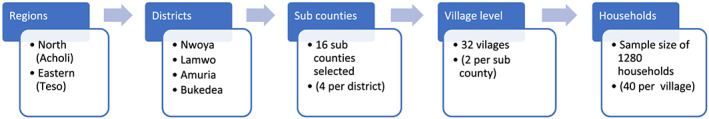
Sampling strategy

Multistage sampling involving mixed methods as shown in Figure 1 was used. Districts were selected based on historical perspective of insurgency and consultations done with local opinion leaders. The objective was to come up with two districts representing areas with more households potentially land tenure insecure and land tenure secure in each subregion. In Acholi, Nwoya and Lamwo districts were purposively selected to represent districts experiencing relatively higher incidence of land tenure insecurity and land tenure security, respectively. In Teso, Amuria and Bukedea districts were purposively selected to represent districts with higher incidence of land tenure insecurity and land tenure security, respectively. In the second sampling stage, two subcounties were randomly selected per district, and in each subcounty, one village chosen following simple random sampling.

The final sampling stage was done at household level to identify households with WRA and especially those with children 6–59 months. The exact household sample size was calculated using Fisher's formula:

$$n=\frac{t^{2}p\left(1-p\right)}{m^{2}},$$

where *n* is the required sample size, *t* is the confidence level at 95% (standard value of 1.96), *p* is the estimated proportion of children under 5 years in the respective four districts with regards to the total population, *m* is the margin of error at 5% (standard value of .05; Magnani, 1997). The total number of respondents (households) was calculated at 1,283 households but was rounded to 1,280, indicating 40 households in each of the 32 randomly selected villages.

To ensure questions targeting the situation 20 years ago are captured and also to ensure households with women of reproductive age are captured; the household selection criteria was to have at least 80% of the sampled households having both a WRA and a child 6–59 months, and at least 10% have at least one household member 40 years and above. Therefore, in all the villages, households having both children 6–59 months and WRA were listed, and another list was generated for households with family members above 40 years of age. Systematic random sampling was used to obtain 1,280 households of which 1,122 (88%) had women 15–49 years. Data were collected by trained enumerators using tablets with a structured questionnaire entered in open data kit.

### 2.2 Description and measurement of selected outcome variables

#### 2.2.1 Access to land and land tenure perceptions

To establish level of access to land, variables such as size of land accessible to the household (farmed, fallowed, and range), type of ownership, and its use for two seasons 2017A (January–July) and 2017B (August–December) were established. Respondents were asked 13 questions (Appendix A) that required them to rate their perception on land tenure insecurity/security given different circumstances. Both land insecurity contributing and land insecurity reducing circumstances formed the set of questions that required nonmultiple selection response following the ordinal scale on level of agreement (1 = *agree*; 2 = *not sure*; 3 = *disagree*). Using factor analysis, (Supporting information), the scores on land tenure insecurity were derived from the set of responses. However, a dichotomous distinction was derived from the degree of insecurity at seven levels (1 *= very insecure;* 2 *= moderately insecure;* 3 *= insecure;* 4 *= indifferent;* 5 *= secure;* 6 *= moderately secure;* 7 *= very secure*), and generally, Levels 1–3 were considered relatively insecure, and Levels 5–7 were considered relatively secure, whereas farmers in Level 4 were left out of the analysis sample (Supporting information). The respondent was required to select a level based on the overall evaluation of responses on a set of 12 contextual question (Appendix A).

#### 2.2.2 Measurement of agrobiodiversity using plant and animal species diversity

In this study, species diversity is considered as a count of the number of different species available in the communities and accessed by the household for use as food. This includes crops grown; animals reared; wild plants and animals (including insects) trapped, gathered, or hunted; and other food commodities obtained through purchase. Thus, this was measured by summing species that a respondent reported to have accessed during the 12 months preceding the survey. For each species (plant or animal) mentioned, the respondents had to provide details such as its source, prime use, its availability, and, where applicable, the level of production in terms of area or number of trees/animals.

#### 2.2.3 Measurement of dietary diversity

Dietary diversity is a qualitative measure of food consumption that reflects household access to a variety of foods and is a proxy indicator of macro and/or micro nutrient adequacy of the diet of individuals (Food and Agriculture Organization [FAO], 2010). Following the FAO standard guidelines and recommended food categories, individuals meeting the minimum dietary diversity (MDD) is generated.

MDD is a dichotomous indicator of whether women 15–49 years of age have consumed at least 5 out of the defined 10 food groups as per FAO (2016) considering the previous 24‐hr day. The proportion of WRA who achieve the MDD in a population can be used as a proxy indicator for higher micronutrient adequacy, one important dimension of diet quality (FAO, 2016).

### 2.3 Analytical technique

Maximum likelihood estimation of the Poisson family was used in this study. The class of Poisson regression applies for outcomes of count data (Long & Freese, 2001; Greene, 2002) and, hence, was used because dietary diversity is measured as a sum of scores, and characteristically, it takes on discrete nonnegative values ranging from 1 to 10. Particularly, the generalized Poisson (GP) model was preferred to the standard Poisson model that operates under the assumption of equal dispersion, yet based on several studies by Harris, Yang, and Hardin (2012), it is hard to achieve in practice. In situations where the variance is lower than the mean, the data are said to be under dispersed. Modelling under dispersed count data using inappropriate models can lead to overestimated standard errors and misleading inference (Harris et al., 2012). According to Husain and Bagmar (2015), the GP regression has statistical advantages over both standard Poisson regression and negative binomial regression models and is suitable for analysis of count data that exhibit either overdispersion or underdispersion. The GP regression is a generalized event count model that is appropriate for both overdispersed and underdispersed count data (Consul & Jain, 1973; Winkelmann & Zimmermann, 1994). In our case, the distribution of dietary diversity score of WRA exhibited under dispersion. This was also justified by the Akaike information criterion factor that was lower for the GP compared with the standard Poisson regression. The primary equation of the Poisson regression model is shown in Equation (1).

(1) $$\mathrm{lnE}\left(Y=J\right)=x^{\prime }\beta ,$$

where the expectation of the number of foods groups eaten is denoted by *Y* outcome that takes on a set of dietary score integers *J* = {1,2,3,…,*j*} dependent on a vector of explanatory variables *x*′ whose quantitative influence is estimated by a vector of parameters *β*.

The model was operationalized through estimation of GP regression following the expression below showing dietary diversity (*y*_*i*_) of WRA *i*, and *y*_*i*_ is hypothesized to be dependent on a set of explanatory variables (*x*_1_*to x*_*n*_) whose respective effect is quantified by the variable specific parameter (*β*).

(2) $$y_{\mathrm{ik}}=\beta_{0}+\beta_{1}x_{1}+\beta_{2}x_{2}+\ldots +\beta_{n}x_{n}$$

Explanatory variables *x*_1_ to *x*_*n*_ included variables categorized in three sets: household socioeconomic factors, land factors, and location factors. Household characteristics included age of household head and spouse, dependency ratio, education of WRA, and income. Age influences household dietary diversity (Mango, Bryon Zamasiya, Nyikahadzoi, & Siziba, 2014), and this is likely to affect dietary diversity of household individuals and information on traditional feeding practices. However, formal schooling is also important because it enhance one's knowledge on diet quality. Additionally, socioeconomic factors such as income levels and household dependency burden can negatively impact on dietary quality (Bouis, Eozenou, & Rahman, 2011). Wealth is a determinant of dietary diversity (Powell, Kerr, Young, & Johns, 2017). Income is also an indicator for the household standard of living and in this case, is meant to control for poverty following the international poverty line of US$ 1.9/day given income also affects food access.

Additionally, location specific factors were also controlled for because species diversity in an area and isolation away from the food markets can potentially reduce access to a wide range of food choices. Distance from the market was therefore added to control for isolation, whereas the dummy for subregional location not only controls for heterogeneity in agro‐ecological conditions (which also influences variations in species diversity and agricultural system) but also controls for variation in access to several species by inhabitants of Acholi and Teso. Ritzema et al. (2019) showed that crop and livestock diversity influence dietary diversity, but also, Guo et al. (2019) observed that production diversity alone is not enough in explaining dietary diversity. Additionally, although wild vegetables may be consumed in small quantities, they influence intake of cereal staples, manage hunger, and play a central role in household food security for the rural poor (Mavengahama, McLachlan, & Clercq, 2013; Walsh & Rooyen, 2014). In this case, the sum of plant and animal species accessed for food is used as a measure of species diversity for food.

The variable land tenure insecurity is measured as a score generated from principle component analysis. The regression was done in two steps. First, with several factors of which others were eliminated after ascertaining that they caused multicollinearity, and others with *p* values less than 0.1 in order to achieve the most parsimonious model.

Based on measures of central tendencies and proportions, data on characteristics of household, WRA is presented, whereas the regression was done in the spirit of multivariate analysis. Generally, data analysis was done using STATA version 14.

## 3 RESULTS

### 3.1 Household characteristics

Results in Table 1 show that 72% of the WRA in Acholi and Teso were in male‐headed households that is 4% higher than the national estimated average for rural areas of Uganda. The results also show that 60% of the household were in monogamous marriages, a figure close to the national level of 57% as per the Uganda census report (UBOS, 2016). Additionally, 79% of the total households sampled had a child aged 6–59 months, and 86% of the WRA had a child under five. The average age for the household heads was 41 years, whereas WRA had an average age of 30 and an average household size of seven that is greater than the reported national average of five (UBOS, 2016). Household heads and WRA on average had a mean of 6 to 7 years of schooling that is equivalent of only primary level formal education.

**TABLE 1:** Profile of surveyed households and interviewed women of reproductive age (WRA)

Variable	Pooled sample (*N* = 1,279)	WRA (*N* = 1,122)	Insecure (*N* = 197)	Secure (*N* = 1,027)
Categorical variables (presented as %)				
Male‐headed households	72.6	70.6	71.1	73.4
Female‐headed households	27.4	29.4	28.9	26.6
Household size				
1–4 members	29.3	32.0	28.7	32.4
5–8 members	42.0	40.1	42.7	39.2
9–12 members	23.0	22.4	20.8	23.3
12 and above	5.8	5.5	7.9	5.1
Have a child 6–59 months	79.0	86.2	81.7	78.0
Female spouse can read and write	29.4	31.9	24.9	30.0
Marital status of household head				
In a monogamous marriage	60.2	62.8	56.4	61.5
In polygamous marriage	22.1	23.1	22.8	21.4
Previously married (& separated)	6.9	7.0	11.7	5.7
Widowed	9.8	6.2	8.6	10.1
Never married	1.1	0.8	0.5	1.3
Roofing material of main house				
Grass	83.4	84.7	91.4	82.2
Iron sheets	16.6	15.3	8.6	17.8
Continuous variables (presented by means)				
Age of household (HH) head	41.1 (14.80)	38.0 (12.47)	39.4 (13.62)	41.6 (15.15)
Age of WRA (years)	33.5 (13.33)	29.6 (7.81)	31.6 (12.16)	34.1 (13.65)
Household size in 2017	6.7 (3.44)	6.9 (3.40)	6.8 (3.37)	6.7 (3.48)
Number of dependents	3.1 (2.65)	3.3 (2.64)	3.2 (2.64)	3.1 (2.67)
Dependency ratio	1.0 (1.16)	1.1 (1.15)	1.1 (1.24)	1.0 (1.14)
Household head years of schooling	6.6 (3.70)	6.9 (3.52)	6.5 (3.56)	6.6 (3.68)
WRA years of schooling	5.7 (2.64)	6.0 (2.52)	5.6 (2.68)	5.8 (2.6)
Family land size	5.9 (15.64)	5.9 (15.07)	5.6 (18.05)	6.1 (15.45)
Income (US$ per day)	0.19 (0.27)	0.2 (0.27)	0.2 (0.25)	0.2 (0.26)
Time to the market	0.9 (1.32)	0.9 (1.33)	0.8 (1.34)	1.0 (1.31)
Number of species	8.1 (3.24)	8.1 (3.23)	8.3 (3.06)	8.1 (3.29)

*Note.* Values in parenthesis are standard deviations.

### 3.2 Access to land and land tenure insecurity

The findings shown in Table 2 indicate that land size and land use has changed between the present (in 2017) and the past years (before 1997). Average land holding was found to have dropped by 50% from an average of 12 acres to an average of 6 acres. Area of land farmed has also dropped by 50% from an average of about 8 acres to an average of 4 acres. Additionally, land under range (covered by grass, shrubs, and trees, left for grazing of either domestic livestock or wild animals or both) has not changed for Acholi, but it has significantly reduced for Teso (Table 2).

**TABLE 2:** Land size

Category description	Mean acreage of land accessed for farming and other use					
	Pooled sample (*N* = 1279)		Acholi (*N* = 640)		Teso (*N* = 639)	
	Present	Past	Present	Past	Present	Past
Land use (acres)						
Land farmed	4.1	7.5	5.5	11.0	2.6	5.0
Land under fallow	1.2	3.3	2.0	6.1	0.4	1.4
Range land	0.6	0.6	1.0	1.1	0.1	0.2

**FIGURE 2:**
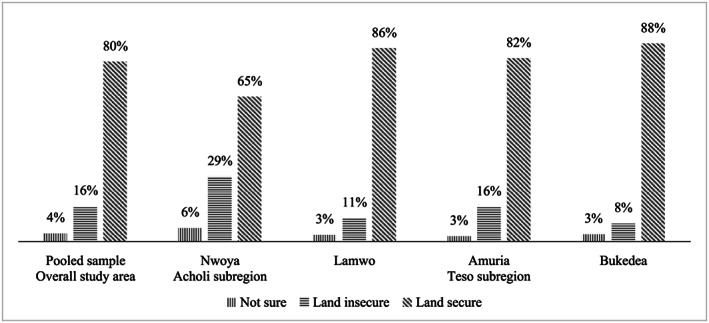
Land tenure insecurity among households in Acholi and Teso

Figure 2 shows that 16% of the households perceived themselves to be land tenure insecure and the level of land insecurity was higher in Acholi (20%) compared with Teso subregion (12%). Generally, 17% of the WRA were living in households with perceived land tenure insecurity. Figure 2 further shows that land tenure insecurity in Acholi was higher in Nwoya District, and in Teso, Amuria District had the highest percentage of land tenure insecure households.

### 3.3 Species diversity

From the 1,279 households, more than 200 different plant and animal species were mentioned as accessible by households for food use. These composed of 55 cultivated plant species, 8 domesticated animal species, and more than 150 wild plant and animal species.

#### 3.3.1 Plant species diversity

More than 35 plant species were recorded as cultivated by the interviewed household members. The most popular cultivated plant species included sorghum and cassava that were cultivated by more than 50% of the households. Other popular food crops included maize, millet, white‐fleshed sweet potato, groundnuts, beans, cowpeas, and sesame seeds (Table 3).

**TABLE 3:** Diversity of plant species cultivated by households in Acholi and Teso subregions

Crop species	Percent of households				
	Overall (*N* = 1,279)	Acholi (*N* = 640)	Teso (*N* = 639)	Insecure (*N* = 197)	Secure (*N* = 1,027)
Maize	46.4	57.8	35.1	52.3	45.0
Millet	20.1	21.6	18.6	20.3	20.3
Sorghum	57.1	65.8	48.4	56.9	58.0
Rice	8.0	11.6	4.4	13.2	6.2
Cooking type banana	1.4	2.3	0.5	2.0	1.3
Cassava	56.0	43.3	68.7	55.8	55.2
Irish potato	0.23	0.5	0.0	0.5	0.2
White flesh sweet potato	14.3	10.3	18.3	16.2	14.1
Orange flesh sweet potato	0.6	0.2	0.9	0.5	0.6
Pumpkin (leaves and fruit)	0.1	0.2	0.0	0.0	0.1
Cocoyam	0.1	0.0	0.2	0.0	0.1
Beans	14.2	24.5	3.9	22.3	12.5
Groundnuts	36.4	35.0	37.7	33.5	36.7
Soybean	3.3	5.2	1.4	5.1	2.6
Pigeon peas	5.7	11.1	0.3	6.1	5.5
Field peas	1.9	3.0	0.8	3.1	1.2
Cowpeas	16.0	3.8	28.2	11.2	17.0
Sesame seed	24.8	48.3	1.3	24.4	25.1
Sunflower	2.7	4.8	0.5	3.1	2.7
Bambara nuts	0.1	0.2	0.0	0.0	0.1
Shea nut tree	0.1	0.2	0.2	0.0	0.2
Green grams	13.0	0.3	25.7	8.6	14.0
Kale	0.0	0.0	0.0	0.0	0.0
Cabbage	0.7	1.4	0.0	1.0	0.7
Eggplant	0.8	1.6	0.0	1.0	0.9
African eggplant	10.2	18.1	2.5	13.2	9.6
Okra	1.1	2.0	0.2	0.5	1.2
Spider plant	0.0	0.0	0.0	0.0	0.0
Cat whiskers	0.1	0.2	0.0	0.0	0.1
Mushroom	0.0	0.0	0.0	0.0	0.0
Tomatoes	1.0	1.6	0.5	1.0	1.0
Onions	0.2	0.3	0.0	0.0	0.2
Spinach	0.0	0.0	0.0	0.0	0.0
Carrots	0.1	0.2	0.0	0.0	0.1
Cucumber	0.0	0.0	0.0	0.0	0.0
Hot pepper	0.1	0.2	0.0	0.0	0.1
Mangoes	3.0	5.8	0.2	6.1	2.4
Pawpaw	1.6	3.3	0.0	2.5	1.5
Avocado	0.6	1.3	0.0	1.0	0.6
Passion fruits	0.0	0.0	0.0	0.0	0.0
Citrus fruits	1.4	2.5	0.3	2.0	1.3
Jack fruit	0.4	0.6	0.2	0.0	0.5
Guavas	0.0	0.0	0.0	0.0	0.0
Desert bananas	0.0	0.0	0.0	0.0	0.0
Watermelon	0.1	0.2	0.0	0.0	0.1
Pineapple	0.0	0.0	0.0	0.0	0.0
Shea fruit	0.1	0.2	0.0	0.5	0.0

#### 3.3.2 Livestock species diversity

Results showed that the animal species most kept by households in Acholi and Teso include cattle (of local breed) followed by goats, chickens, sheep, pigs, and turkeys. A significantly higher percentage of households in Teso kept cattle compared with Acholi, whereas the reverse was true for chickens. Table 4 further shows that Teso had a significantly higher percentage (93%) of households with cattle compared with those of Acholi (14%), and also, 83% of households that considered themselves land tenure secure had cattle, as compared with 24% of households that perceived themselves to be land tenure insecure. The difference in proportion cattle ownership by land tenure secure and insecure households was not statistically different.

**TABLE 4:** Percentage of households with various livestock

Animal species	Overall (*N* = 1,279)	Acholi (*N* = 640)	Teso (*N* = 639)	*χ* ^2^	Insecure (*N* = 197)	Secure (*N* = 1,027)	*χ* ^2^
Cattle	74.2	13.8	92.5	.000	23.5	83.3	.001
Goats	73.4	72.4	73.1	.090	82.4	72.6	.126
Chickens	62.1	82.8	54.8	.052	58.8	61.8	.805
Pigs	10.5	3.5	10.8	.208	17.7	7.8	.233
Sheep	12.9	6.9	14.0	.535	11.8	11.8	.874
Turkeys	4.0	0.0	3.2	.311	0.0	2.0	.555
Rabbits	4.0	0.0	3.2	.311	0.0	2.9	.469
Ducks	2.4	2.4	0.0	.085	0.0	1.0	.677

#### 3.3.3 Wild species diversity

Overall, 69% and 41% of households reported to have gathered some plant species and/or hunted some animal species, respectively. More than 100 wild species that could be used for food were reported by the 1,279 respondents. Approximately 78 animal species, 10 bird species, 14 fish species, 23 fruit species, 13 insect species, and more than 80 wild plant species were reported. It is shown that plant and animal species are the most gathered and hunted (Table 5). Plant species most maintained within their natural habitats were mainly fruits and they included mangoes, oranges, pawpaw, and jackfruit maintained by 19%, 15%, 9%, and 7% of the households, respectively.

**TABLE 5:** Proportion of households accessing the different types of wild species

Species	Overall (*N* = 1,279)	Acholi (*N* = 640)	Teso (*N* = 639)
Type of wild species			
Plant species	69.0	49.4	88.7
Animal species	41.0	71.9	10.0
Insect species	12.2	12.7	11.7
Bird species	8.3	7.8	8.8
Fish species	2.7	3.0	2.4
Number of wild species			
0 wild species	8.7	10.7	6.7
1–2 wild species	69.4	61.1	00.0
3–4 wild species	17.9	22.3	77.6
5–6 wild species	3.5	4.7	13.5
7–8 wild species	0.5	0.9	2.3
9–13 wild species	0.2	0.3	0.0

Also, despite the large diversity in edible wild species, the level of their utilization varies in the two subregions, but utilization of fish, bird, and insect species is generally very low in both Acholi and Teso subregions. Table 5 shows that 61% of households in Acholi gather one to two species from the wild, whereas about 78% of households in Teso gather three to four species from the wild. Thus, households in Teso gathered/hunted more wild species compared with households in Acholi. Generally, the insecure households had better access to wild species compared with households that perceived themselves land tenure secure.

#### 3.3.4 Crop grown and overall species diversity

The results further reveal that irrespective of the subregion, most households grew three to four different crops, and particularly, 66% of the households in Teso compared with 55% of households in Acholi grew three to four crops. On account of perceived land tenure insecurity vis‐a‐vis land tenure security, it was found that for both Acholi and Teso subregions, households that perceived themselves to be secure had more crop diversity compared with those that perceived themselves land tenure insecure (Table 6). For instance, 55% of the land tenure insecure households grow three to four crops compared with 61% of the land secure households. Additionally, 59% of land insecure households had access to five to nine wild species compared with 57% of the land secure counterparts.

**TABLE 6:** Crop and overall species diversity among land tenure insecure and land tenure secure households

No. of species (categories)	Percentage of households						
	Pooled sample			Acholi		Teso	
	Overall (*N* = 1,279)	Insecure (*n* = 197)	Secure (*n* = 1,027)	Insecure (*n* = 124)	Secure (*n* = 483)	Insecure (*n* = 73)	Secure (*n* = 544)
Number of crops grown							
1–2 crops	34.1	37.1	33.6	36.3	36.0	38.4	31.4
3–4 crops	60.5	55.3	61.3	51.6	54.7	61.6	67.3
5–6 crops	5.0	7.6	4.6	12.1	8.3	0.0	1.3
7–8 crops	0.4	0.0	0.5	0.0	1.0	0.0	0.0
Number of total species accessed							
1–4 species	11.9	9.6	12.4	7.3	10.8	13.7	13.8
5–9 species	57.2	59.4	56.8	53.2	54.2	69.9	59.0
10–14 species	27.3	27.9	27.2	35.5	30.6	15.1	24.1
15–19 species	3.4	3.1	3.4	4.0	3.7	1.4	3.1
≥20 species	0.2	0.0	0.3	0.0	0.6	0.0	0.0

Generally, 57% of the households had access to a total of five to nine species (Table 6). A comparison of species diversity for households that considered themselves land tenure insecure and those that considered themselves secure reveals that access is not significantly different for the pooled sample. However, there are significant differences in the percentage of (the land tenure insecure vis‐a‐vis the land tenure secure) households within the subregions. For instance, in Teso, 15% of land tenure insecure households accessed 10 to 14 species compared with 24% of their counterparts who were land tenure secure. Generally, it is revealed that households access a limited number of species, which may predicate a lower number of food groups consumed.

### 3.4 Dietary diversity

#### 3.4.1 Diversity of diets consumed by WRA

This section presents results on dietary diversity of WRA. Out of a sample size of 1,279, 88% of the women fell within the age range of 15–49, and thus, a study sample size of 1,122 was used to assess dietary diversity parameters of WRA.

Table 7 shows that the most common food groups consumed were cereals, roots, and tubers with more than 89% of all the WRA having consumed foods from these groups. Other popular food groups included leafy vegetables (80%), meat/fish (55%), pulses/legumes (55%), and nuts and seeds (43%). The findings also indicate that consumption of cereals, roots, tubers, and bananas; pulses/legumes; and nuts and seeds was not statistically different among WRA from households that perceived themselves to be land tenure insecure versus the land tenure secure. Consumption of vegetables and fruits (either vitamin A rich or not) was significantly higher among households that considered themselves land tenure insecure compared to the land tenure secure. This is explained by the fact that most households considering themselves to be land insecure were also observed to be living in areas closer to conservation areas and could have had better access to foods from wild habitats compared with their counterparts.

**TABLE 7:** Food groups consumption by WRAs in 24 hr preceding the survey

Food group	Percent of women of reproductive age consuming the foods					
	Overall (*N* = 1,122)	Acholi (*N* = 606)	Teso (*N* = 516)	Insecure (*N* = 189)	Secure (*N* = 888)	χ^2^
Cereals & RTBs	92.1	89.1	95.5	92.6	92.0	.936
Dark green leafy vegetables	80.4	71.8	90.5	88.4	78.8	.010
Other vegetables	79.1	70.5	89.3	85.2	77.9	.081
Meats & fish	55.1	44.2	67.4	56.1	54.5	.855
Pulses/legumes	55.0	53.5	57.0	57.1	54.3	.476
Nuts and seeds	43.8	42.4	45.5	41.3	44.3	.699
Vitamin A‐rich fruits & vegetables	14.6	16.8	12.2	19.6	13.4	.055
Milk and milk products	13.0	9.6	16.5	11.6	13,3	.379
Other fruits	11.5	14.7	8.0	19.1	9.6	.000
Eggs	1.7	1.5	1.9	1.1	1.8	.742

Abbreviations: RTBs, Roots, Tubers, Bananas; WRA, women of reproductive age.

#### 3.4.2 WRAs that met MDD requirement

Despite the observed relatively high percentage of women consuming starchy staples, legumes/pulses, nuts/seeds, green leafy vegetables, and meats and fish, considering the overall number of women of reproductive age, 47% were not meeting their MDD score. The findings also showed that a significantly higher proportion of women in Acholi subregion (57.1%) were not meeting MDD as compared with those not meeting the same in Teso (35.3%). Additionally, WRA who did not meet the MDD ate more of starchy staples (cereals, roots, tubers, and bananas) and vegetables and a lesser percentage ate pulses, nuts/seeds, dairy, meats, eggs, vitamin A‐rich vegetables and fruits. Women who met their MDD requirement ate most of the foods in the food basked (Table 8). However, for all categories, consumption of milk and milk products, vitamin A‐rich vegetables and fruits, and eggs was low. This is attributed to the high price often placed on such products to the extent that even when produced at household level, they are highly regarded for income generation rather than consumption.

**TABLE 8:** Food group consumption by WRAs who met MDD and those who did not meet MDD in the last 24 hr preceding the survey

No.	Food group	Percentage of women		
		Never met MDD (*N* = 528)	Met MDD (*N* = 594)	*p* value
1	Cereals & white tubers	84.3	99.0	.000
2	Pulses	32.4	75.3	.000
3	Nuts and seeds	24.4	61.1	.000
4	Milk and dairy products	6.3	18.5	.006
5	Meat, organ meats, poultry, & fishes	36.9	70.9	.000
6	Eggs	0.4	2.9	.001
7	Dark green leafy vegetables	60.7	97.9	.000
8	Vitamin A‐rich vegetables and fruits	8.3	20.4	.000
9	Other vegetables	57.6	98.3	.000
10	Other fruits	5.7	16.8	.001

Abbreviations: DD, dietary diversity; MDD, minimum dietary diversity; WRA, women of reproductive age.

### 3.5 Drivers of dietary diversity

The results presented are in the spirit of a generalized Poisson multivariate regression of *DD*_*i*_ and a set of regressors (*x*) for WRA, and the results are presented in two panels, standard regression coefficients and after computation of marginal effects.

The results show that dietary diversity of WRAs exhibits a positive correlation with a male‐headed household, age of household head, species diversity, daily disposable income, and education of WRA, whereas increase in WRA age, dependency ratio, land tenure insecurity, and distance to the market negatively correlate with dietary diversity (Table 9).

**TABLE 9:** Multivariate regression results on drivers of diversity in foods eaten by women of reproductive age

Explanatory variable	Coefficient	Marginal effect
Sex of HH head (1 = male, 0 = female headed)	0.0224 (0.0459)	0.1004 (0.2060)
Age of HH head (log)	0.2586^**^ (0.0786)	1.1600^**^ (0.3544)
Age of mothers (years)	−0.0036 (0.0031)	−0.0162 (0.0139)
Dependency ratio	−0.0446^*^ (0.0173)	−0.2000^**^ (0.0778)
Size of family land (acres in 2018)	−0.0012 (0.0017)	−0.0053 (0.0078)
Number of species accessed	0.0403^***^ (0.0065)	0.1806^***^ (0.0296)
Land insecurity factor (score)	−0.0592^**^ (0.0208)	−0.2656^**^ (0.0935)
Disposable income per day (USD)	0.0046 (0.0709)	0.0206 (0.3182)
Distance to the market (minutes)	−0.0344^*^ s(0.0156)	−0.1542^**^ (0.0699)
Schooling of WRA (years)	0.0101 (0.00774)	0.0452 (0.0347)
Respondent location/AEZ (0 = Acholi; 1 = Teso)	0.3823^***^ (0.0442)	1.7147^***^ (0.2003)
Constant	0.8717^***^ (0.2572)	
Atanh delta	0.2404 (0.0177)	
Delta	0.2359 (0.0167)	
LR test of delta = 0: *χ* ^2^(1) = 570.76, *p* = .000		
Wald *χ* ^2^ = 161.86, *p* > *χ* ^2^ = .000		
Observations (*N*) = 1,072		

*Note.* All the variables listed in the table were the ones included in the final multivariate Poisson regression analysis. Robust standard errors in parentheses.

*** *p* < .01.

** *p* < .05.

* *p* < .1.

After controlling for the various factors including age, sex of household head, land size, household income, and distance to the market, we find that a unit increase in the number of species accessed for food is likely to increase dietary diversity of WRA by 18% (*p* < .000), other factors notwithstanding. Additionally, the perception of being land tenure insecure has a likelihood of reducing diversity of diets consumed by women of reproductive age by 26% (*p* < .05).

### 3.6 Limitations of the study

The study tried to control for variation in species diversity due to geographical and cultural differences; however, variation in soil quality, which is an important determinant of what a farmer can produce, was not controlled for basing on the scope of the study. Additionally, this was a cross‐sectional study, where data were collected at only one point in time.

## 4 DISCUSSION

### 4.1 Agrobiodiversity and dietary diversity

Although Uganda lacks a complete record of the status of its agrobiodiversity, Uganda is ranked among the top 10 most biodiverse countries in the world, endowed with great diversity of animal and plant species (National Environment Management Authority [NEMA], 2016). There is an estimation of about 1,400 indigenous plant species in Uganda with more than 200 species of noncultivated edible plants and 75 species of indigenous edible fruits. In addition, about 55 exotic species of plants both fruits and vegetables have been recorded (NEMA, 2016). Despite the relatively high agrobiodiversity found in Uganda and in the study area, findings of this study show only a handful of them (11 plant species and five animal species) are being used by more than 10% of the study population. The lack of optimum utilization of agrobiodiversity has also been reported in Uganda generally, where many of the indigenous 1,400 plant species have not been exploited, and the loss of agrobiodiversity due to lack of utilization, conservation, mechanized agriculture, and population pressure is estimated to be at an average of 10% per year (NEMA, 2016). Most households cultivated between three and four different plant species and reared between one and two livestock species. Despite the reported number of species, the popular plant species included maize, sorghum, cassava, and all starchy staples, whereas the popular livestock included chicken, cattle, and goats mainly local breeds, and according to the respondents, these are mainly used in acquisition of income. Agriculture is the main source of food and income for most Ugandans in rural areas, and if biodiversity is lost in agricultural systems and yet many have access to limited sources of plant and animal species for food, the options to make their diets healthier and sustaining resilient food systems become adversely compromised. This further explains the findings of this study, which indicate that a unit increase in species diversity leads to a 18% increase in dietary diversity of WRA.

### 4.2 Dietary diversity of WRA

Different foods and food groups are good sources for various macronutrients and micronutrients, so a diverse diet best ensures nutrient adequacy. Considering women, their dietary diversity has been shown to be significantly associated with reduced anaemia, and it is also significantly associated with reduced low birth weight and preterm birth (Zerfu, Umeta, & Baye, 2016). Therefore, dietary diversity for women of reproductive age is very important. Although dietary diversity is globally recommended, it is especially important among populations in low‐ and middle‐income countries where diets are mainly based on starchy staples and where micronutrient deficiencies are highly reported (FAO, 2016). In most parts of Uganda, diets are mainly based on starchy staples such as banana, maize, cassava, and sorghum, depending on the geographical region. Findings from this study show that although half of the women of reproductive age were meeting their minimum dietary requirements, consumption of cereals, roots, and tubers was high, and about half was reported consuming meats and fish but less than 10% were consuming eggs, milk, and milk products. The observed positive relationship between species diversity and dietary diversity supports reports that higher food self‐sufficiency, nutritional functional diversity, and dietary diversity scores are positively correlated with higher crop and animal species richness (Luna‐González & Sørensen, 2018).

### 4.3 Relationship between land tenure, agrobiodiversity, and dietary diversity

Secure access to enough land is an important means of achieving food security in poor agrarian land‐scarce societies, and strong tenure security for landowners stimulates investment and efficient land use (Holden & Ghebru, 2016). Holden and Ghebru further reported that women put more emphasis on household food security than their husbands and that having joint land certification/ownership resulted in women being more influential in crop choice and land rental decisions, and this was related to better consumption and nutrition outcomes of children and family in general. This explains the observed relationship between land tenure insecurity and dietary diversity of WRA in this study. The negative correlation between land tenure insecurity and dietary diversity is attributed to the likelihood of it, limiting investment and production on the land more so, the type of animal species reared, and crops planted. It may also affect what is maintained/conserved on the land much as not domesticated. In that regard, the number of species available for home consumption is negatively affected, leading to a significant downward impact on the dietary diversity of the WRAs.

## 5 CONCLUSION

From the findings that dietary diversity of WRA is consistently, positively correlated with species diversity and negatively with land tenure insecurity, interventions with an aim to increase species diversity can deliver positive dividends for food and nutrition security, whereas land policy reforms and interventions that strengthen land tenure security for both men and women are more likely to contribute positively to biodiversity and dietary diversity. This would lead to improved food and nutrition security of vulnerable communities in rural areas.

## ACKNOWLEDGEMENTS

The Drivers of Food Choice (DFC) project was implemented by team of staff from Trócaire Uganda and Bioversity International (Uganda office). The implementing partners are indebted to all the parents and children that participated in this study. All the support from the team of kind field guides, village and/or clan leaders, and district leaders that offered their kind support and advice to the Trócaire and Bioversity International implementing team. This research was funded by the DFC Competitive Grants Program,which is under the U.K. Government's Department for InternationalDevelopment and the Bill & Melinda Gates Foundation and managedby the University of South Carolina, Arnold School of Public Health,USA; and the views expressed do not necessarily reflect the various partners' official policies.

## CONFLICTS OF INTEREST

The authors declare that they have no conflicts of interest.

## CONTRIBUTIONS

BE designed the study, initiated the publication, and coordinated and led the improvement of the manuscript during review process. RMA developed the protocols, performed data collection, data analysis, played a major role in putting together the results and the methodologies section, and revised the manuscript during the review process. GK provided technical backstopping during the study and provided great input during development of this manuscript especially the methods and analysis work and has been involved in revision of the manuscript during its review. ID played a major role in leading development of protocols for collecting data on land tenure in/security and actively engaged in the development of the manuscript through regular feedback on methodology and results. MB actively engaged in development of protocols for collecting data on land tenure in/security and actively engaged in the development of the manuscript through regular feedback on Introduction, Methods, Results, and Discussion with special focus on land related issues.

## Supporting information

Data S1. Supporting Information

## APPENDIX A LAND INSECURITY QUESTIONS

**Table:**

	The perception of households in relation to their land tenure stability/instability	
QN	On your main parcel, please state your level of acceptance whether you agree, indifferent or disagree with the following statement in regard to family land (1 = *agree*; 2 = *not sure*; 3 = *disagree*)	Choice
1	Generally, how do you feel about the security of your land given this is where you have lived for years?	
2	This land has been owned and controlled by me and/or my family for many years	
3	There are no disputes in my family or the community in relation to my land	
4	We are hindered in how we manage our land because there is uncertainty about who controls it	
5	Because of uncertainties surrounding our land, we are not able to use it the way we would like to	
6	My family & I regularly make long‐term decisions about how to use our land because we feel very secure on that land	
7	There is no value in improving or developing my/our land due to many issues surrounding this land and I may not have this land in 5‐ or 10‐years' time	
8	The land is on customary land and so is secure for a very long time to come	
9	Our land is on customary land & thus has uncertainty about family controlling it in the long term	
10	There has been a lot of land grabbing in this community and my land is also not safe.	
11	My husband's/wife's relatives are always trying to get our land, so it has caused a land conflict?	
12	I expect that I & my family/children will continue to farm land for a very long time to come	
13	The big challenge we have in my household is land dispute between the parents & the children	

*Note.* 1 = *very insecure*; 2 = *moderately insecure*; 3 = *insecure*; 4 = *indifferent*; 5 = *secure*; 6 = *moderately secure*; 7 = *very secure.*
