# Supplementary material for: Relationships between land tenure insecurity, agrobiodiversity, and dietary diversity of women of reproductive age: Evidence from Acholi and Teso subregions of Uganda
Source: Matern Child Nutr. 2020 Dec 21;16(Suppl 3):e12965. doi: 10.1111/mcn.12965 (PMC7752126; doi:10.1111/mcn.12965)
Supplement: Supplementary file 1 — Data S1. Supporting Information [file MCN-16-e12965-s001.docx]

# Factor analysis outputs

## Eigen values

Output 1 shows that factor 1 and factor 2 have a combined variance of 6.91 which is actually a proportion of 0.997 of the total variances explained by factor analysis. Thus, cumulatively, factor 1 and factor 2 explain 99.7% of the variance in the described land tenure characteristics. All the 13 components combined explain 100% of the variance in the variables/characteristics of land tenure insecurity factor. This shows that estimating factor 1 and factor 2 can be taken as sufficient for the analysis.

**Results output 1:**

**Rotated variable matrix**

Generally, factor one had a high correlation with 8 out of the 12 characteristics and thus was considered the land tenure insecurity factor. Land tenure insecurity factor was more associated with the elements in question 3 to 8, 10 and 12. The threshold for correlation was set at 0.50 (50%) to obtain the rotated factor loadings (See output).

**NOTE: QN1 was asked last because it required a respondent to give a response based on the evaluation of Q2-Q13**

| **Question** | **Element definition** |  |
| --- | --- | --- |
| 2 | Ownership & control of the land for many years | -0.4453 |
| 3 | No land dispute on the land | -0.6533 |
| 4 | Limited land management due to Uncertainty on who controls the land | 0.7923 |
| 5 | Difficulty in using the land because of uncertainty | 0.7849 |
| 6 | Ability to make long term decisions on how to use the land because of security | -0.5141 |
| 7 | No value in land development due to land issues and long-term uncertainty | 0.6936 |
| 8 | Security in the long run because of customary tenure | -0.5980 |
| 9 | Feeling uncertain on land control in the long run because of customary tenure | 0.4718 |
| 10 | Feeling unsafe because of land grabbing in the community/area | 0.5647 |
| 11 | Land conflict with relatives | 0.4911 |
| 12 | Expectation that will farm the land for a very long time in the future | -0.6421 |
| 13 | Land dispute between the parent(s) and the children | 0.4525 |
| 1 | General feeling of security on land given occupancy for many years |  |

**Output 2**

**Alpha reliability factor**

**Measure of sampling adequacy = 0.9016**

**Variable clustering**

Score plot for factor 1
